# 

**Published:** 1921-07-02

**Authors:** 


July 2, 1921] [Price Twopence

### The Hospital

The Workers' Newspaper of Administrative Medicine and Institutional Life, Administration, National Insurance and Health.

CONTENTS

The Conference at Manchester ... ... ... 225
The Government and the Hospitals ... ... 226
The Manchester Conference of 1911 .. ... 226
Notes of the Week ... ... ... ... ... 227
A Chance for a Joint Appeal—Alexandra Day—The Saturday Funds and Trade Depression—Hospital Sunday—The Hospital Pageant—London Secretaryships—Maintenance Charges Deferred—Encouragement for Maternity Hospitals—Convalescence in Lodgings—A Patient's Letter-Book—Hospital Stamps at Leicester—Guardians and the Middle Classes.
X-Rays and Cancer ... ... ... ... ... ... 229
What Recent Announcements Really Mean.
The Notification of Measles ... ... ... ... ... 229
The Anthrax Order ... ... ... ... ... ... 230
The Federation of Medical and Allied Societies ... 230
The Public Well-Being ... ... ... ... ... 231
The Economic Waste of Illness—Crime and Unemployment.
Home Helps Schemes ... ... ... ... ... ... 232
Scheme to Raise £100,000 ... ... ... ... 233
An Active Building Programme at Leicester.
The Folkestone Health Congress ... ... ... 233
Nurse Registration in Scotland ... ... ... 234
Draft Rules Now Available.
The Matrons' and Sisters' Department ... ... 235
Fever Nurses and the General Register.
Round the Hospitals ... ... ... ... ... 236
The College of Nursing (Limited by Guarantee) 238
The Annual Meeting and Conference.
Scientific, Social and Economic Questions.
English and Scottish Matrons in Conference ... vi
The League of Remembrance (1914-1919) ... viii
Forthcoming Events ... ... ... ... ... viii
Passing Events and Topics ... ... ... ... viii

